# Safety of Completion Thyroidectomy for Initially Misdiagnosed Thyroid Carcinoma

**DOI:** 10.5041/RMMJ.10249

**Published:** 2016-07-28

**Authors:** Gangiti Kranthikumar, Nusrath Syed, Hemantkumar Nemade, Satish Pawar, L. M. Chandra Sekhara Rao, T. Subramanyeshwar Rao

**Affiliations:** Department of Surgical Oncology, Basavatarakam Indo-American Cancer Hospital and Research Institute, Hyderabad, India

**Keywords:** Completion thyroidectomy, hypoparathyroidism, recurrent laryngeal nerve palsy, revision thyroid surgery, thyroid cancer

## Abstract

**Introduction:**

Completion thyroidectomy is defined as the surgical removal of the remnant thyroid tissue following procedures of less than total or near-total thyroidectomy. Whether thyroid reoperations are associated with an increased complication risk is controversial.

**Objective:**

A retrospective analysis was done of patients undergoing completion thyroidectomy for cancer of the thyroid who had undergone surgery elsewhere for solitary thyroid nodule. The incidence of surgical complications in these patients after reoperation was investigated in this study.

**Material and methods:**

The study included a total of 53 patients who had undergone thyroid lobectomy for a solitary nodule as initial surgery elsewhere and were referred to our institute for completion thyroidectomy when the histopathology revealed malignancy.

**Results:**

There were 53 patients, 43 females and 10 males. Their mean age was 34.7±12.12 years (range 19–65 years). After initial surgery, the histopathology revealed papillary carcinoma in 46 patients (86.8%), follicular carcinoma in 7 (13.2%). Fourteen out of 53 patients had recurrent laryngeal nerve palsy after initial surgery (26.4%). None of the patients had clinical hypocalcemia after the first surgery. One or more parathyroid glands were identified and preserved in 52 patients (98.1%) in the process of completion thyroidectomy. No patient had additional recurrent nerve injury at the second surgery. The mean serum calcium value preoperatively was 8.96±0.39 mg/dL, and six months after surgery serum calcium was 8.74±0.56 mg/dL. Mean follow-up was 18 months. Transient hypoparathyroidism occurred in 24.5% patients. Five patients were lost to follow-up. Permanent and symptomatic hyperparathyroidism occurred in eight patients (16.67%).

**Conclusions:**

Completion thyroidectomy is a safe and appropriate option in the management of well-differentiated thyroid cancer. It removes disease on the ipsilateral and contralateral side of the thyroid and carries a low risk of recurrent laryngeal nerve damage, but a higher risk of permanent hypoparathyroidism.

## INTRODUCTION

Solitary thyroid nodule is one of the common presentations in a thyroid clinic. The usual evaluation for the solitary thyroid nodule includes fine-needle aspiration cytology (FNAC), which in many cases is inconclusive. This mandates hemithyroidectomy followed by completion thyroidectomy in the majority of the cases of well-differentiated carcinoma. Ultrasonography and FNAC has led to a 35%–75% decrease in the number of patients requiring surgery.^1^–^4^ As a result, there has been a substantial increase in the proportion of malignant lesions in surgical cases. Completion thyroidectomy is traditionally believed to increase complication rates, but many studies contradict this belief.^5^–^13^

We analyzed our data to discover whether completion thyroidectomy increased the incidence of recurrent nerve palsy and parathyroid gland-related complications. Furthermore, we investigated the data in terms of ipsilateral and contralateral sides (i.e. the previously operated side and the side untouched in the previous surgery, respectively).

## MATERIAL AND METHODS

Data were obtained for patients who underwent surgery for solitary thyroid nodule elsewhere, including from nursing homes with surgeons not trained in head and neck oncology, and were referred to our institute for completion thyroidectomy after a postoperative histological diagnosis of cancer.

Some patients were referred specifically because of the complications during previous surgery, such as recurrent laryngeal nerve palsy. Medical records and details of 53 such patients who met the above criteria and underwent completion thyroidectomy between June 2012 and June 2014, with a mean follow-up of 18 months and a minimum of one year, were collected.

All patients underwent thorough clinical examination and neck ultrasonography, thyroid hormone assessment, and indirect laryngoscopy before the second operation. All the histological slides and blocks of the first surgery were reviewed by the pathology experts. Only patients with confirmed diagnosis of papillary thyroid carcinoma with tumor >2 cm, and all follicular carcinomas, were enrolled in the study of patients who underwent completion thyroidectomy. Serum calcium levels were determined preoperatively and postoperatively after 48 hours. All symptomatic patients were started on oral and intravenous calcium supplementation, and asymptomatic patients with hypocalcemia signs were started on oral calcium supplementation. All patients underwent vocal cord assessment after the second surgery at the time of discharge. Postoperatively all patients with papillary and follicular carcinomas were referred for radioiodine ablation. Thereafter patients were followed every six months with serum thyroid-stimulating hormone, serum thyroglobulin, serum antithyroglobulin, and serum calcium.

### Surgical Technique

A single dose of antibiotic was administered just before the incision. The previous incision was revised and extended if necessary. After elevating subplatysmal flaps and retracting strap muscles in the midline, both sides of the thyroid or thyroid bed and the isthmus were carefully evaluated for any thyroid tissue. Meticulous dissection was performed, sometimes approaching the thyroid gland laterally from the strap muscles. Surgical loupes were used during surgery. A nerve monitor was not used. The superior laryngeal nerve, recurrent laryngeal nerve (RLN), and parathyroid glands were identified before resecting thyroid tissue. Where required, appropriate neck dissection was done. The central compartment was dissected only for locally advanced disease or when preoperative ultrasonography or intraoperative assessment revealed suspicious nodes in that region. At the end of the procedure if the parathyroid tissue appeared devascularized it was implanted in the sternocleidomastoid muscle.

## RESULTS

There were 53 patients, 43 women and 10 men. Their mean age was 34.7 years (range 19–65 years) (Table 1). The period between initial and second surgery ranged from 10 days to 150 days, with a median of 60 days. Mean operating time was 68 min. After initial surgery, the histopathology revealed papillary carcinoma in 47 patients (86.8%) and follicular carcinoma in 6 (13.2%). Fourteen out of 53 patients had recurrent laryngeal nerve palsy after initial surgery (26.4%). One or more parathyroid glands were identified and preserved in 52 patients (98%); parathyroids could not be identified in one patient. No patient had additional recurrent nerve injury at the second surgery. The mean serum calcium value preoperatively was 8.96±0.39, and postoperative serum calcium was 8.74±0.56 after six months. No patient required readmission. Mean follow-up was 18 months. Transient hypoparathyroidism occurred in 13 (24.5%) patients. Five patients were lost to follow-up. Permanent and symptomatic hypoparathyroidism occurred in eight patients (8/48 patients, 16.67%) (Tables 2 and 3).

**Table 1:** Patient Demographics.

Patient and Surgery Details	*n*
**Age Group**	
<20	2
20–40	38
40–60	10
>60	3
**Sex**	
Male	10
Female	43
**Surgery**	
Subtotal thyroidectomy, B/L MRND [including B/L paratracheal (central compartment) node dissection]	12
Subtotal thyroidectomy	13
Lobectomy, B/L paratracheal (central compartment) node dissection	10
Hemithyroidectomy only	4
Lobectomy only	14
Pathology	
Papillary carcinoma	47
Follicular carcinoma	6

B/L, bilateral; MRND, modified radical neck dissection.

**Table 2:** Complications.

Complications	*n* (%)
**After First Surgery**	
RLN palsy	14 (26.4%)
Hypocalcemia	0
**After Second Surgery**	
Additional RLN palsy	0
Lost to follow-up	5 (9.4%)
Transient hypoparathyroidism	13/53 (24.5%)
Permanent hypoparathyroidism	8/48 (16.67%)
Mortality	0

RLN, recurrent laryngeal nerve.

**Table 3:** Second Surgery Type and Hypothyroidism.

Surgery	Cases of Permanent Hypoparathyroidism (*n*)
Subtotal thyroidectomy, B/L MRND [including B/L paratracheal (central compartment) node dissection]	2
Subtotal thyroidectomy	1
Lobectomy, B/L paratracheal (central compartment) node dissection	3
Hemithyroidectomy only	1
Lobectomy only	1

B/L, bilateral; MRND, modified radical neck dissection.

There was no mortality and no chylous leak. There was no additional RLN injury, and the mean pre- and post-completion thyroidectomy serum calcium levels were 8.96±0.39 mg/dL and 8.74±0.56 mg/dL, respectively. No patient developed wound infection or hematoma requiring exploration.

Thirteen patients had temporary hypocalcemia, whereas eight patients had permanent and symptomatic hypoparathyroidism. Five patients in the postoperative course of six months regained a normal serum calcium level without need of supplementation. Eight patients had persistent hypocalcemia and remained symptomatic, requiring oral calcium supplementation. Five of these patients had undergone bilateral paratracheal nodal dissection.

## DISCUSSION

A completion thyroidectomy is required when papillary or follicular thyroid carcinoma is diagnosed postoperatively in patients undergoing partial thyroidectomy. It facilitates removal of the residual thyroid tissue/disease, permits screening and ablation of metastatic disease with radioactive iodine, and allows for thyroglobulin level monitoring, thereby eliminating the risk of recurrence of the tumor and prolonging survival.

In addition, it is reported that presence of multifocal tumor is associated with a high risk of lymph node metastasis.^11^ Studies have reported residual tissue malignancy rates of 33% to 44% after the completion surgery.^5^–^10^ It was reported to be as high as 78% in Levin’s series.^12^ Several authors have concluded that completion thyroidectomy is safe; associated morbidity and timing had no impact on development of complications.^5^–^13^ The rate of the recurrent laryngeal nerve palsy varies from 0% to 4%.^5^–^13^

Although some studies recommend that completion thyroidectomy should be performed either before scar tissue development or after clinical remission of scar tissue, edema, and inflammation, recent evidence shows that timing of surgery has no effect on morbidity.^10^

Recurrent laryngeal nerve (RLN) injury can occur after thyroid surgery. The rate of RLN injury, mostly transient, ranges from 0.5% to 5%.^14^ The risk is more important in patients who undergo reoperative thyroid surgery and in patients with thyroid cancer or hyperthyroidism.^12^ A meticulous surgical technique can lower the postoperative morbidity if precise operative rules are respected. Though the literature describes very low rates of recurrent laryngeal nerve injury during the primary thyroid surgery, in our series it was higher, probably because the majority of the cases were performed in nursing home setups by surgeons not trained in head neck oncology.

In our study there was no additional RLN injury in completion thyroidectomy. We used surgical loupes for meticulous dissection and identification of the nerves and parathyroid. A nerve monitor was not used in this study. Completion thyroidectomy was shown to be a fairly safe procedure, which carries a low incidence of complications and also facilitates further management and follow-up with radioactive iodine. There was no recurrent laryngeal nerve palsy in the study by El-Zohairy and Zaher.^15^ Similar results were noted with a 0% incidence of RLN injury when completion thyroidectomy was necessary for the treatment of thyroid malignancy, and it was effective for diagnosing and removing occult disease in the remaining thyroid by Kupferman et al.^5^

In another study of 647 patients, conducted by Gulcelik et al. who compared groups for complications, there were no significant differences except temporary hypocalcemia between completion thyroidectomy and total thyroidectomy for differentiated thyroid carcinoma.^16^ The routine use of intraoperative neuromonitoring seems not to reduce the incidence of RLN during revision thyroid surgery, at least in the setting of a tertiary referral center.^17^

Hypocalcemia after thyroidectomy is the most common postoperative complication, with a reported incidence from 0.5% to even 50% of the operated patients.^18^

Transient hypoparathyroidism is said to be present when oral/intravenous calcium supplements are required for less than six months after surgery, and permanent hypoparathyroidism if hypocalcemia symptoms last more than six months or when the requirement for oral calcium/intravenous supplements lasts longer than six months.

In a large series of 5,000 patients undergoing bilateral thyroid surgeries the overall incidence of transient and permanent hypoparathyroidism was 7.3% and 1.5%, respectively. Extent of resection and surgical technique had a greater impact on the rates of permanent postoperative hypoparathyroidism than thyroid pathologic condition.^19^ Other series have reported an incidence of temporary hypoparathyroidism of 0% to 14%, and a permanent hypoparathyroidism incidence around 2% to 8%.^5^–^10^,^13^,^20^–^22^

With improvements in surgical technique and experience, complication rates of thyroidectomy performed for benign or malignant diseases are reduced. In spite of the improvement in surgical experience, temporary RLN palsy and hypoparathyroidism are the main complications in completion thyroidectomies, which need special attention.^16^

Hypocalcemia occurred more frequently when neck dissection was combined with total thyroidectomy (60%) than without it (17%) (*P*<0.005). The incidence of hypocalcemia was higher after central, than lateral, neck dissection.^23^ In a series by Roh et al. the incidence of temporary hypocalcemia was 46.3%, and permanent hypocalcemia 4.9%, in patients undergoing central compartment reoperation for recurrent/persistent differentiated thyroid cancer.^24^ Other series did not reveal increased morbidity following central compartment dissection.^25^,^26^

In our study, transient hypoparathyroidism occurred in 13 (24.5%) patients. Five patients were lost to follow-up. Permanent and symptomatic hypoparathyroidism occurred in eight patients (8/48 patients, 16.67%).

We considered all patients for radioiodine ablation as the tumor size in previous histopathology reports could not be verified, and in a significant number of patients we found residual thyroid tissue in the previously operated thyroid bed.

## CONCLUSION

Completion thyroidectomy is a safe and appropriate option in the management of well-differentiated thyroid cancer. It removes disease on ipsilateral and contralateral sides of the thyroid and carries a low risk of recurrent laryngeal nerve damage, but a higher risk of permanent hypoparathyroidism. The incidence of hypocalcemia was higher after central than lateral neck dissection. With improvements in surgical technique and knowledge, complication rates of completion or revision thyroidectomy are reduced.

## Abbreviations

FNAC: fine-needle aspiration cytology
RLN: recurrent laryngeal nerve
